# Albumin for patients with acute large vessel occlusive stroke undergoing endovascular therapy-2 (ARISE-2): protocol for a randomised controlled trial

**DOI:** 10.1093/esj/aakag119

**Published:** 2026-10-05

**Authors:** Zilin Xu, Yuanyuan Liu, Yanbo Huang, Yuzhang Bei, Yong He, Yanling Li, Hao Wang, Lingling Meng, Le Gao, Yan Li, Yuhang Cao, Lan Liu, Jinyue Li, Xunming Ji, Chuanjie Wu

**Affiliations:** Department of Neurology, Xuanwu Hospital, Capital Medical University, Beijing, China; Department of Neurology, Xuanwu Hospital, Capital Medical University, Beijing, China; Department of Neurology, People's Hospital of Huazhou, Maoming, China; Department of Neurology, Liuyang Jili Hospital, Liuyang, China; Department of Neurology, Liuyang Jili Hospital, Liuyang, China; Department of Neurology, Shenqiu County People's Hospital, Zhoukou, China; Department of Neurology, Linyi City People Hospital, Linyi, China; Department of Neurology, Zhongmu County People's Hospital, Zhengzhou, China; Department of Neurology, Xuanwu Hospital, Capital Medical University, Beijing, China; Department of Neurology, Xuanwu Hospital, Capital Medical University, Beijing, China; Department of Neurology, Xuanwu Hospital, Capital Medical University, Beijing, China; Beijing Institute of Brain Disorders, Capital Medical University, Beijing, China; National Stroke Administration Office, Xuanwu Hospital Capital Medical University, Beijing, China; Department of Neurology, Xuanwu Hospital, Capital Medical University, Beijing, China; Beijing Institute of Brain Disorders, Capital Medical University, Beijing, China; Institute of Clinical Research, Chinese Academy of Medical Sciences/Peking Union Medical College, Beijing, China; Department of Neurology, Xuanwu Hospital, Capital Medical University, Beijing, China; Beijing Institute of Brain Disorders, Capital Medical University, Beijing, China

**Keywords:** albumin, acute large vessel occlusive stroke, acute ischemic stroke, endovascular therapy, China

## Abstract

**Background:**

Endovascular therapy is the standard of care for eligible patients with acute ischemic stroke due to LVO. However, nearly half of patients remain functionally dependent even after successful reperfusion. Albumin is a biologically plausible adjunctive cerebroprotective therapy and has shown neuroprotective effects in preclinical studies, but the clinical benefit of albumin combined with EVT remains uncertain.

**Study design:**

Albumin for patients with acute large vessel occlusive stroke undergoing endovascular therapy-2 (ARISE-2) is an investigator-initiated, prospective, multicenter, randomised, open-label, controlled trial with blinded endpoint assessment (PROBE design). Eligible participants will be randomly assigned in a 1:1 ratio to receive either EVT plus intravenous 25% albumin at 0.5 g/kg (maximum dose, 37.5 g) initiated within 60 min after randomisation and then repeated once daily on days 2–4, or EVT alone. The initial target sample size is 1192 participants (596 per group). A prespecified adaptive sample size re-estimation will be performed after approximately 50% of participants have completed the 90-day follow-up, allowing the total sample size to be increased, but not reduced, to a maximum of 1788 participants. This trial aims to evaluate the efficacy and safety of albumin administered in combination with EVT in patients with acute ischemic stroke caused by anterior circulation LVO.

**Study endpoints:**

The primary outcome is a favourable functional outcome at 90 days, defined as a mRS score of 0–2, or return to the premorbid mRS for participants with a prestroke mRS score of 3–4.

**Summary:**

The ARISE-2 trial is designed to evaluate the efficacy and safety of moderate-dose albumin combined with EVT in participants with acute ischemic stroke caused by anterior circulation LVO. The trial will provide evidence on whether albumin can improve functional outcomes as an adjunctive cerebroprotective strategy in patients undergoing reperfusion therapy.

**Trial registration:**

ClinicalTrials.gov: NCT07263308.

## Graphical Abstract

**Graphical Abstract:**
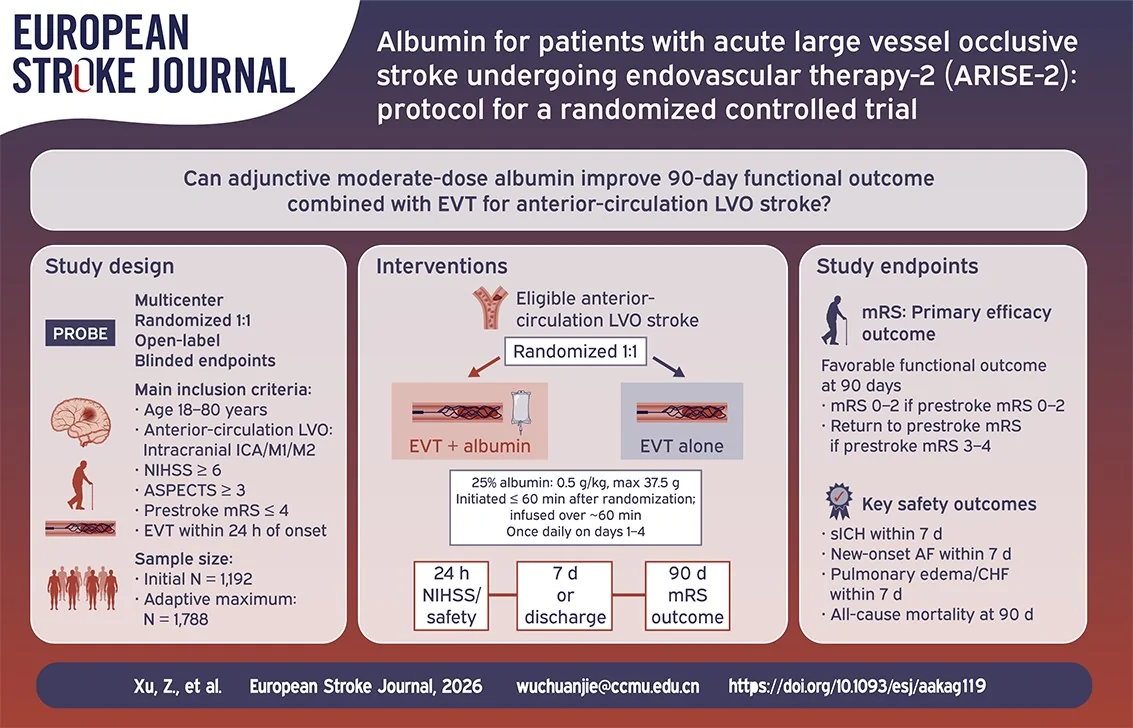

## Introduction and rationale

Acute ischemic stroke caused by LVO is a major cause of death and long-term disability worldwide. Endovascular therapy has become the standard of care for eligible patients, including selected patients treated in an extended time window.^1–3^ Nevertheless, functional recovery remains suboptimal in about half of patients despite successful reperfusion.^4^ This mismatch between vessel reopening and clinical recovery, commonly referred to as futile recanalisation, and ischemic injury before reperfusion as well as ischemia–reperfusion injury after reperfusion may be important contributors.^4^ Therefore, this underscores the need for adjunctive therapies that target tissue-level injury before, during and after reperfusion.

Albumin is an endogenous plasma protein synthesised primarily by the liver. Preclinical animal studies suggest that albumin can reduce infarct volume and improve neurological function. Although its precise mechanisms in ischemic stroke have not been fully established, albumin has been shown in animal studies to exert cerebral protective effects through antioxidant, anti-inflammatory, endothelial-stabilising and microcirculatory mechanisms.^5–7^ The ALIAS (Albumin in Acute Stroke) trial series, which comprised several pilot and randomised controlled trials, investigated the efficacy of albumin in patients with acute ischemic stroke. The ALIAS Pilot Trial evaluated the tolerability of albumin in acute ischemic stroke and provided preliminary evidence that albumin was more effective in patients who received tPA reperfusion therapy.^8^^,^^9^ ALIAS Part 1 found that in patients who received tPA reperfusion therapy, albumin increased the proportion with a favourable outcome from 36.6% to 46.7%, but the trial was suspended early due to safety concerns.^10^^,^^11^ Unfortunately, the definitive ALIAS Part 2 trial failed to confirm a clinical benefit of albumin in patients with acute ischemic stroke and was associated with an increased incidence of intracerebral haemorrhage and pulmonary oedema.^12–14^ Overall, these studies nevertheless laid the groundwork for the field and suggest that reducing albumin-related adverse effects and combining albumin with reperfusion therapies may guide future clinical trials of albumin in acute ischemic stroke.

In the proof-of-concept ARISE (albumin for patients with acute large vessel occlusive stroke undergoing endovascular therapy) trial, we administered a moderate dose of albumin (0.5 g/kg daily for 4 days) in combination with EVT, which represents a contemporary and more effective reperfusion strategy. The results suggested that albumin reduced infarct expansion without increasing cardiovascular or pulmonary complications.^15^ However, the trial was not powered to detect differences in long-term functional outcome. Therefore, the ARISE-2 trial was designed to evaluate the efficacy and safety of a moderate dose of albumin administered in combination with EVT, compared with EVT alone, in patients with acute anterior circulation LVO.

## Methods

### Study design

ARISE-2 is an investigator-initiated, prospective, multicentre, randomised, open-label, controlled trial with blinded endpoint assessment (PROBE design). The trial is designed in accordance with the Declaration of Helsinki and the SPIRIT 2025 recommendations.^16^ The protocol has been approved by the Ethics Committee of Xuanwu Hospital, Capital Medical University and the trial is registered at ClinicalTrials.gov (NCT07263308). Participants will be recruited from approximately 50 stroke centres in China. Written informed consent will be obtained from the participant or a legally authorised representative before randomisation.

### Patient population

Eligible participants are patients aged 18–80 years with acute ischemic stroke caused by anterior circulation large vessel occlusion, defined as occlusion of the intracranial internal carotid artery or the M1 or M2 segment of the middle cerebral artery, who are planned to undergo EVT within 24 h of symptom onset. Key eligibility criteria include a baseline NIHSS score of at least 6, an Alberta Stroke Program Early CT Score (ASPECTS) score of at least 3 and a prestroke mRS score of 0–4.

Key exclusion criteria are conditions that may increase the risk of albumin-related volume load, including recent or current heart failure, acute myocardial infarction, hemodynamically significant arrhythmia; heart failure related to valvular disease within the previous 6 months, oxygen-dependent acute or chronic pulmonary disease. Full eligibility criteria are provided in Table 1. Laboratory eligibility criteria, including haemoglobin and liver function, will be assessed using routine admission laboratory results obtained as part of standard clinical care. No additional study-specific blood sampling or repeat laboratory testing is required for trial screening, and laboratory assessment and preparation for reperfusion therapy will proceed in parallel to avoid treatment delay.

**Table 1:** Inclusion and exclusion criteria for the ARISE-2 trial.

Inclusion criteria	Exclusion criteria
1. Age between 18 and 80 years;2. Acute ischemic stroke with indications for EVT within 24 h of onset; 3. Anterior circulation acute ischemic stroke, with acute occlusion of the responsible vessel located in the intracranial segment of the internal carotid artery or the M1 or M2 segment of the middle cerebral artery; 4. NIHSS score ≥ 6; 5. ASPECTS ≥3 points; 6. mRS score ≤ 4 before stroke onset; 7. Written informed consent was provided by the patients or their legal representatives.	1. Evidence of intracranial haemorrhage (ICH, SAH, epidural haemorrhage, acute or chronic subdural hematoma) on the baseline CT or MRI scan; 2. History of congenital or acquired bleeding disorders, coagulation factor deficiency diseases, thrombocytopenic diseases, etc. 3. Anticipated difficulty in completing EVT due to vascular tortuosity; 4. Known severe allergy (more severe than skin rash) to contrast agents uncontrolled by medications; 5. Pregnancy, breastfeeding or planning to get pregnant in 6 months; 6. An episode or exacerbation of congestive heart failure from any cause in the past 6 months; 7. History of heart valve disease complicated by congestive heart failure within the past 6 months; 8. Cardiac surgery with thoracotomy (eg, coronary artery bypass grafting or valve replacement surgery) within the past 6 months; 9. Acute myocardial infarction in the past 6 months; 10. Signs or symptoms of acute myocardial infarction upon admission, including electrocardiographic findings; 11. Elevated serum troponin concentration upon admission (>0.1 μg/L); 12. Acute arrhythmia (including any tachycardia or bradycardia) with hemodynamic instability (systolic blood pressure < 100 mm Hg) upon admission; 13. Acute or chronic lung diseases requiring long-term or intermittent oxygen therapy; 14. Findings on physical examination of any of the following abnormalities: (1) Jugular venous distension (jugular venous pulsation > 4 cm above the sternal angle); (2) Resting tachycardia due to congestive heart failure (heart rate > 100 per/min); (3) Third heart sound; (4) Abnormal hepatic jugular venous reflux; (5) Pitting oedema of the lower extremities attributable to congestive heart failure or without apparent cause; (6) Rales in both lungs; (7) Or evidence of pulmonary oedema, pleural effusion, or pulmonary vascular redistribution on chest X-ray; 15. Severe chronic anaemia (haemoglobin < 75 g/L); 16. Known recent or current serum creatinine exceeding 1.5 times the upper limit of normal, or eGFR < 60 mL/min; 17. Refractory hypertension that is difficult to control by medication (defined as SBP > 220 mmHg, or DBP > 110 mmHg); 18. Serum ALT or AST is greater than 1.5 times the upper limit of the normal value. 19. History of albumin allergy or known allergy to albumin; 20. Patients with severe mental disorders or dementia who are unable to cooperate in completing informed consent and follow-up content; 21. The expected survival time is less than 90 days (such as comorbidity with malignant tumours or severe systemic diseases, etc.); 22. Patients who have participated in other interventional clinical studies within 30 days before randomisation or are currently participating in other interventional clinical studies; 23. The investigator believes that the subject has other conditions, making it unsuitable for participating in this trial.

Abbreviations: EVT = endovascular treatment; NIHSS = National Institute of Health Stroke Scale; ASPECTS = Alberta Stroke Program Early CT Score; mRS = modified Rankin scale; ICH, intracerebral haemorrhage; SAH = subarachnoid haemorrhage; eGFR = estimated glomerular filtration rate; SBP = systolic blood pressure; DBP = diastolic blood pressure; ALT = alanine aminotransferase; AST = aspartate aminotransferase.

### Randomisation and allocation concealment

Eligible participants will be randomly assigned in a 1:1 ratio to either EVT plus albumin (albumin group) or EVT alone (control group). Treatment allocation will be based on a minimisation procedure to balance assignment according to prespecified baseline factors, including centre, age (18–64 vs 65–80 years), sex (female vs male), baseline NIHSS score (<15 vs ≥15), baseline ASPECTS (<8 vs ≥8) and intravenous thrombolysis (yes vs no). Treatment allocation will be concealed via a centralised, web-based randomisation system. Treatment assignments will be revealed only after participants are irreversibly randomised, preventing prediction or manipulation. Outcome assessors will remain blinded throughout the trial.

### Treatment and intervention

#### Albumin group

In addition to EVT, 25% human serum albumin (Shanghai Raas Blood Products Co., Ltd.) will be administered intravenously at 0.5 g/kg, with a maximum dose of 37.5 g. The first infusion will be initiated as soon as feasible after randomisation, within 60 min after randomisation and before the end of the EVT procedure defined as arterial access closure. There is no requirement for albumin administration to be initiated before groin puncture, and no separate interval from diagnostic imaging to initiation of albumin infusion is prespecified. The infusion will be delivered through a dedicated intravenous line over approximately 60 min. The same dose will be repeated once daily on days 2, 3 and 4. Albumin administration may overlap with, but must not delay, EVT.

#### Control group

Participants will receive EVT without albumin administration.

#### Endovascular therapy and medical management in both groups

EVT will be initiated as soon as possible after randomisation. The choice of endovascular technique will be at the discretion of the treating interventionist in accordance with local practice and current guidelines. Other aspects of acute stroke management and secondary prevention will be provided according to current guidelines in both groups.^1^^,^^17^^,^^18^

### Follow-up and data collection

Follow-up assessments will be conducted at post-EVT, 24 h, 7 days or discharge (whichever occurs first) and 90 days after randomisation. In-hospital assessments will include neurological evaluation, vital signs, concomitant treatments, laboratory and imaging data as clinically indicated and adverse events. All assessments of the primary and secondary clinical outcomes will be performed by trained, certified assessors who are masked to treatment allocation (Table 2).

**Table 2:** ARISE-2 study schedule.

	Trial period					
	Screening and enrolment		Post-randomisation			Close-out
Timepoint	−24 h–0 h	0	Post-EVT	24 h	7d	90
**Screening**						
** Demographic data**	X					
** Medical history**	X					
** Vital Sign**	X					
** Physical examination**	X					
** Laboratory examinations**	X					
** CT/MRI**	X					
** MRA/CTA/DSA**	X					
** ASPECTS**	X					
** NIHSS**	X					
** Pre-stroke mRS**	X					
**Enrolment**						
** Eligibility criteria**	X					
** Informed consent**	X					
** Randomisation**		X				
**Intervention**						
** EVT + albumin**		EVT + 0.5 g/kg of albumin IV qd × 4 days				
** EVT**		EVT alone				
**Assessment**						
** eTICI**			X			
** NIHSS**				X	X	
** mRS**						X
** EQ-5D-5L**						X
** BI**						X
** AE/SAEs**			X	X	X	X
** Concomitant medication**			X	X	X	X

Abbreviations: MRA = magnetic resonance angiography; CTA = computed tomography angiography; DSA = digital subtraction angiography; ASPECTS = Alberta Stroke Program Early Computed Tomography Score; NIHSS = National Institutes of Health Stroke Scale; mRS = modified Rankin Scale; EVT = endovascular treatment; eTICI = expanded Thrombolysis in Cerebral Infarction; BI = Barthel index; AE = adverse event; SAE = serious adverse event.

### Study outcomes

Following a protocol amendment on April 12, 2026, in response to the 2026 AHA/ASA guideline update,^1^ the primary efficacy outcome was revised from mRS 0–2 at 90 days to a composite definition of favourable functional outcome at 90 days. The endpoint is defined as meeting either of the following criteria: (1) 90-day mRS score of 0-2 for participants with a prestroke mRS score of 0–2; (2) return to prestroke functional level, defined as a 90-day mRS score less than or equal to the prestroke mRS score, for participants with a prestroke mRS score of 3–4. The secondary efficacy outcomes are NIHSS score at 24 h and at 7 days or discharge; excellent functional outcome, defined as an mRS score of 0–1 at 90 days; ordinal distribution of mRS scores at 90 days; Barthel Index ≥95 at 90 days; and EQ-5D-5L at 90 days.

Safety outcomes include symptomatic intracranial haemorrhage within 7 days, defined according to the Heidelberg Bleeding Classification; incidence of newly onset atrial fibrillation within 7 days; incidence rate of newly onset pulmonary oedema or congestive heart failure within 7 days and all-cause mortality at 90 days.

### Data and safety monitoring board

An independent Data and Safety Monitoring Board (DSMB), whose members are not otherwise involved in the conduct of the trial, will monitor participant safety and trial integrity. The DSMB will review recruitment, protocol adherence, accumulating safety data and serious adverse events at prespecified intervals. The DSMB may recommend continuation, protocol modification, temporary suspension, or termination of the trial to the steering committee.

### Patient and public involvement

Patients and the public were not involved in the development of the research question, selection of outcome measures, trial design, recruitment, or conduct of the trial. Two patients and their caregivers will participate in discussions during manuscript preparation and, after publication, in planning and activities for dissemination of the trial findings. After trial results publication in peer-reviewed journals, results will be disseminated to all participants through communication with trial clinicians and the media.

### Sample size estimate

The sample size calculation was based on the unpooled variance formula for two independent proportions, with a two-sided type I error rate of 0.05 and 80% power. Based on the event rates observed in the ARISE trial (53.9% in the albumin group vs 45.6% in the control group), the required sample size is 566 participants per group. Allowing for an anticipated 5% loss to follow-up or withdrawal, the initial recruitment target is 596 participants per group, for a total of 1192 participants.

Given the residual uncertainty in the true event rate and treatment effect for the primary outcome, a prespecified adaptive sample size re-estimation will be performed at the interim analysis. The interim analysis will be conducted when approximately 50% of participants (*n* = 596) have completed the 90-day follow-up and will be conducted by an independent statistician who is not involved in trial conduct and reviewed by the DSMB. Sample size re-estimation will be performed using a prespecified promising-zone approach based on conditional power for the primary outcome.^19^ Depending on interim results, the trial will continue with the original sample size or increase recruitment up to a maximum of 1,788 participants. This maximum is derived to detect a more conservative treatment effect corresponding to 80% of the preceding observed effect, thereby providing a robust safeguard against overoptimism while maintaining operational feasibility. No sample size reduction or unblinding of treatment allocation will occur.

### Statistical analysis

The primary efficacy analysis will be performed in the intention-to-treat population, defined as all randomised participants according to their assigned treatment group. A per-protocol analysis will be conducted as a sensitivity analysis. The safety analysis will be performed in the safety population, defined as all participants in the control group and all participants in the albumin group who receive any amount of albumin.

Missing values from loss to follow-up will be handled via multiple imputation by chained equations, incorporating baseline covariates and available intermediate outcomes. A worst-case sensitivity analysis will also be conducted.

The primary outcome will be analysed using a modified Poisson regression model with robust error variance, adjusted for including but not limited to age, sex, baseline NIHSS, baseline ASPECTS and intravenous thrombolysis. Results will be reported as adjusted risk ratios with 95% confidence intervals.

Other binary secondary outcomes will be analysed using a modified Poisson regression model with robust error variance. The ordinal distribution of the 90-day mRS will be analysed using ordinal logistic regression. If the proportional odds assumptions is violated, a generalised odds ratio as the prespecified alternative will be considered to use. Continuous outcomes will be analysed using linear regression. If the assumption is seriously violated, quantile (median) regression will be used in sensitivity analyses. All regression models will include the same prespecified covariates as the primary analysis, unless convergence issues arise.

Prespecified subgroup analyses will be performed to explore potential heterogeneity of the treatment effect according to the following clinical and procedural characteristics: age (18–64 vs 65–80 years), sex (female vs male), baseline NIHSS score (<15 vs ≥15), baseline ASPECTS (<8 vs ≥8), intravenous thrombolysis (yes vs no), stroke subtype (large-artery atherosclerosis vs cardioembolism vs other or undetermined aetiology), final eTICI grade (2b vs 2c/3), occlusion site (intracranial internal carotid artery vs M1 vs M2 segment of the middle cerebral artery) and prestroke mRS score (0–2 vs 3–4). Treatment-by-subgroup interactions will be assessed to explore potential heterogeneity of treatment effects. No adjustment for multiple comparisons is planned for secondary outcomes or subgroup analyses.

All tests will be two-sided, and *P* < .05 will be considered statistically significant. All analyses will be conducted using R software (R Foundation for Statistical Computing) and SAS software (SAS Institute Inc.).

### Blinding, quality control and adjudication

To minimise assessment bias in this open-label trial, ARISE-2 will implement a PROBE design with blinded endpoint assessment. The 90-day mRS and other clinical endpoints will be assessed through structured interviews by certified assessors who are masked to treatment allocation and not involved in clinical care. All mRS interviews will be audio- or video-recorded for central adjudication. Clinical endpoint events and safety events will be adjudicated by an independent central adjudication committee according to prespecified criteria. All data will be captured via an electronic data capture system. In compliance with Good Clinical Practice (GCP) standards, 100% source data verification for key endpoints and logic checks will be performed, with all queries resolved prior to database lock under the oversight of an independent data management team.

## Discussion

Although EVT has substantially improved vascular recanalisation and functional outcomes in acute ischemic stroke caused by large vessel occlusion, a considerable proportion of patients remain disabled despite successful reperfusion. This persistent gap highlights the need for adjunctive cerebroprotective strategies that complement vessel reopening by targeting tissue-level injury after recanalisation. The Stroke Treatment Academic Industry Roundtable has emphasised that previously developed cytoprotective agents should be reconsidered when they have shown robust protective effects in preclinical models and an acceptable safety profile in prior clinical studies, particularly in the reperfusion era.^20^ In this context, ARISE-2 is designed to determine whether albumin, administered as an adjunct to EVT, improves 90-day functional outcome in patients with acute anterior circulation large vessel occlusion stroke.

Recent reperfusion-era trials have strengthened the rationale for combining cytoprotective therapies with recanalisation treatment. In TASTE-2 (Edaravone dexborneol versus placebo on functional outcomes in patients with acute ischaemic stroke undergoing endovascular thrombectomy), edaravone dexborneol increased the likelihood of functional independence at 90 days compared with placebo among patients with acute ischemic stroke undergoing endovascular thrombectomy within 24 h, without an excess of serious adverse events.^21^ Similarly, the BAST (Efficacy and Safety of Butylphthalide in Patients with Acute Ischemic Stroke: A Randomized Clinical Trial) trial provided evidence that butylphthalide improved 90-day functional outcome in patients with acute ischemic stroke receiving intravenous thrombolysis and/or EVT.^22^ In addition, a phase 1b/2a clinical trial showed that ApTOLL administered before EVT was safe and associated with potentially meaningful reductions in infarct-related injury, mortality and disability at 90 days compared with placebo.^23^ Collectively, these studies support the concept that adjunctive cerebroprotection may improve outcomes in patients receiving reperfusion therapy by mitigating reperfusion injury, microvascular dysfunction, inflammation and tissue-level damage.

Albumin is a biologically plausible adjunct to mechanical reperfusion. Its potential mechanisms include maintenance of colloid oncotic pressure, attenuation of cerebral oedema, antioxidant activity mediated partly by free thiol groups, endothelial and blood–brain barrier stabilisation, improvement of microvascular flow and modulation of inflammatory and thrombo-inflammatory responses within reperfused tissue.^5–7^ Clinical studies conducted before the modern EVT era established the feasibility of albumin administration but failed to demonstrate a definitive clinical benefit, partly because high-dose systemic albumin was associated with cardiopulmonary safety concerns and was tested in broad, heterogeneous acute stroke populations.^8–14^ In the reperfusion era, the earlier ARISE trial tested a moderate-dose intravenous regimen of albumin, 0.5 g/kg daily for 4 days, in patients undergoing EVT and suggested reduced infarct expansion without an apparent increase in pulmonary oedema or congestive heart failure.^15^ However, whether this tissue-level signal translates into improved long-term functional outcome remains uncertain. Complementing the intravenous strategy, a pilot trial further demonstrated the feasibility and safety of local intra-arterial albumin delivery after successful mechanical thrombectomy, supporting continued investigation of albumin-based strategies in the EVT setting.^24^

ARISE-2 aims to address this evidence gap by evaluating moderate-dose albumin in patients with anterior circulation large vessel occlusion stroke undergoing EVT. The regimen of 0.5 g/kg for 4 consecutive days was selected to preserve the potential cerebroprotective effects of albumin while reducing the volume-related risks observed with high-dose albumin in earlier trials. Although the earlier ARISE trial did not show an increased risk of pulmonary oedema or congestive heart failure with this regimen, its sample size was limited and it was not designed to provide definitive evidence on clinical outcomes. Therefore, ARISE-2 will provide evidence on whether albumin has an adjunctive therapeutic role in EVT-based treatment for anterior circulation large vessel occlusion stroke and will further clarify its safety profile in the acute reperfusion setting. However, the trial is limited to patients with anterior circulation large vessel occlusion who are planned for EVT; future studies will be needed to determine whether any observed treatment effect can be generalised to other stroke subtypes, posterior circulation occlusion, or patients who do not undergo EVT.

## Data Availability

No participant-level data are available at the protocol stage. After completion of the trial and publication of the primary results, deidentified participant-level data and relevant trial documents will be made available upon reasonable request to the corresponding author, subject to approval by the steering committee and in accordance with applicable ethical approvals, institutional policies and data protection regulations.
